# Venlafaxine-induced thrombocytopenia: A case report and literature review

**DOI:** 10.1097/MD.0000000000043413

**Published:** 2025-07-25

**Authors:** Shi Lijun, Ma Zhongrui, Wei Li, Feng Lei, Jiang Wei, Yu Xia, Pan Yaning

**Affiliations:** a Department of Hematology, Chengdu Fifth People’s Hospital, Chengdu, China; b Department of Psychosomatic Medicine, Chengdu Fifth People’s Hospital, Chengdu, China; c Geriatric Diseases Institute of Chengdu, Chengdu, China; d Department of Training Section, Chengdu Fifth People’s Hospital, Chengdu, China.

**Keywords:** drug-induced thrombocytopenia, SNRI essential thrombocythemia, thrombocytopenia, venlafaxine

## Abstract

**Rationale:**

Drug-induced thrombocytopenia (DITP) is an adverse drug effect mediated by drug-dependent antibodies. Although several cases of thrombocytopenia induced by antidepressants and antianxiety drugs have been reported at home and abroad, reports of venlafaxine hydrochloride-induced thrombocytopenia are rare.

**Patient concerns:**

In this study, we report a case of an anxiety patient who developed severe thrombocytopenia and subcutaneous bleeding after 1 week of treatment with a venlafaxine-containing regimen.

**Diagnoses:**

DITP was considered after other secondary factors were excluded.

**Interventions:**

The patient’s platelet count returned to normal after treatment with glucocorticoids combined with recombinant human thrombopoietin, and antianxiety drugs without venlafaxine were administered for continued treatment.

**Outcomes:**

Three months after discharge, repeated routine blood monitoring indicated that the platelet count remained normal.

**Lessons:**

In combination with the cases and related literature, we would like to emphasize that venlafaxine may rarely cause thrombocytopenia, and clinicians should pay attention to and inform patients of rare adverse reactions that may occur if they encounter venlafaxine in their clinical practice. Once DITP is considered, the drug should be discontinued and relevant treatment measures should be taken. Based on this case and a review of the literature, we suggest that all cases with myelodysplastic tumor characteristics should undergo screening for BCR/ABL genes or Ph chromosomes to exclude chronic myeloid leukemia to mitigate the risk of misdiagnosis and ensure timely initiation of appropriate treatment.

## 1. Introduction

Drug-induced thrombocytopenia (DITP) is a relatively common and potentially serious adverse effect of therapy with numerous structurally unrelated drugs.^[1]^ Several drugs have been implicated in the development of DITP.^[2]^ Among psychotropic drugs, drugs that can cause thrombocytopenia are tricyclic antidepressants, monoamine oxidase inhibitors, sertraline in selective serotonin reuptake inhibitors (SSRIs), citalopram, α2-adrenergic receptor antagonists, serotonin 1, serotonin 2 receptor antagonists mianserin and norepinephrine, and the specific serotonin antidepressant mirtazapine.^[3]^ Venlafaxine is a serotonin (5-HT) and norepinephrine reuptake inhibitor; however, there are few reports on venlafaxine-induced thrombocytopenia, and we report a case of possible venlafaxine-induced thrombocytopenia. Platelet count returned to normal after platelet infusion and glucocorticoid treatment combined with recombinant human thrombopoietin treatment. Antianxiety treatment was continued outside the hospital, and platelet reexamination was in the normal range; therefore, combined with the analysis of relevant literature, we would like to emphasize that in clinical practice, attention should be paid to the rare adverse reactions that venlafaxine may cause thrombocytopenia, and once found, the suspected drugs should be stopped in time and intervention measures should be taken.

## 2. Case report

A 47-year-old male patient was admitted to the hospital with poor sleep for 1 year and aggravation for 1 month on July 26, 2023. One year before admission, the patient had no obvious cause of poor sleep, accompanied by a difficulty falling asleep, falling asleep for more than 30 minutes, shallow sleep surface after falling asleep, and easily waking up early. During the day, the patient felt inattention and memory loss, and often experienced restlessness, tension, worry, numbness, and other discomfort due to pressure at work. He sought medical treatment at a local hospital, and successively took “Alprazolam,” “Esazolam,” “Zolpidem tartrate,” and other drugs to improve sleep, and the aforementioned symptoms were relieved. One month prior to admission at our hospital, the patient underwent physical therapy at a local hospital due to “plantar fasciitis.” As the attending physician informed him that the disease might cause poor recovery and even have sequelae such as difficulty walking, the patient became more nervous, worried, and fearful on the night after returning home, with restlessness, palpitations, chest tightness, and fatigue, which was accompanied by generalized sweating after each subsequent episode. Three days before admission, he underwent treatment at the outpatient department of our hospital. After treatment with “Zolpidem tartrate, tandospirone citrate,” his symptoms did not improve significantly, and was admitted to the hospital for further treatment. The patient was previously healthy, with no history of thrombocytopenia or drug allergy. Family history was negative, and physical examination showed no abnormalities. Blood routine examination on the day of admission showed: hemoglobin 147 g/L, white blood cells 8.75 × 10^9^/L, neutrophils 5.61 × 10^9^ L, platelets 139 × 10^9^/L. Thyroid function, immune set, liver and kidney function electrolyte, infection index, chest computed tomography and coronary computed tomography angiography showed no abnormalities. A score of 4 was obtained on the Hamilton Anxiety Scale, and a score 6 on the Hamilton Depression Scale. After exclusion of physical diseases, the diagnosis was considered as generalized anxiety disorder.

**Figure 1.**
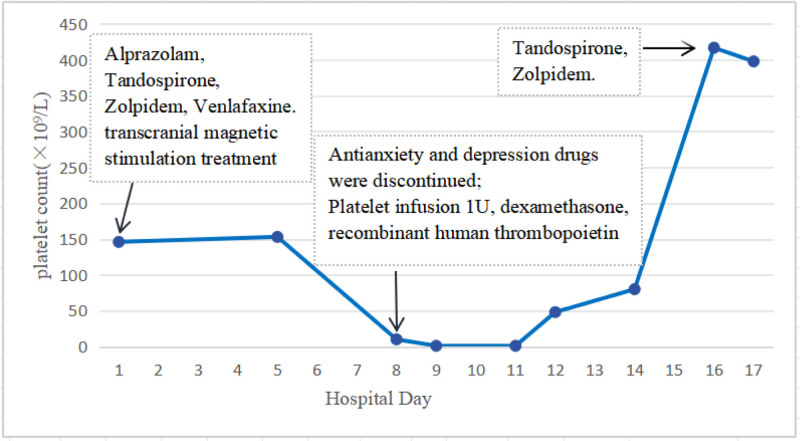
Depiction of patient’s platelet count trend over time in response to exposure to and removal of venlafaxine.

The patient was administered Alprazolam 0.4 mg once/night, tandospirone citrate capsule 15 mg 3 times/d, Zolpidem tartrate tablet 5 mg once/night, and venlafaxine hydrochloride sustained release tablet 75 mg once/d. At the same time, repeated transcranial magnetic stimulation treatment was administered, and the aforementioned symptoms were significantly alleviated. On the eighth day of treatment, scattered petechiae appeared in both the lower limbs, and the platelet count dropped to 10 × 10^9^/L after reexamination. To rule out pseudothrombocytopenia, blood routine examination was performed after replacing the ethylene diamine tetraacetic acid tube twice, which indicated that the platelet count was still low, regardless of pseudothrombocytopenia. Considering the possibility of drug-related thrombocytopenia, the patient was transferred to the department of hematology for further treatment. After transfer, the patient was reexamined and the platelets were 10 × 10^9^/L. Infection index, immune index, and bone marrow puncture examination showed no abnormalities. Based on the patient’s medical history and other examination results, the diagnosis was drug-related thrombocytopenia. Antianxiety and depression drugs were discontinued, platelet support was transfused, dexamethasone 40 mg/d × 4 days, recombinant human thrombopoietin injection 15,000μ, subcutaneous platelet treatment once/day, and platelet count was 417 × 10^9^/L after 8 days of treatment (Fig. 1). After discharge, tandospirone citrate capsule 15 mg 3 times a day, Zolpidem tartrate tablet 5 mg once/night, and venlafaxine hydrochloride sustained-release tablets were not readministered. After repeated outpatient follow-ups, the patient’s mental symptoms were stable and the platelet examination was 398 × 10^9^/L. Finally, it was considered that the thrombocytopenia might have been caused by venlafaxine.

## 3. Discussion

DITP is one of the most common types of thrombocytopenia observed in outpatient clinics. Epidemiological studies conducted in Europe and the United States have shown that the annual incidence of DITP is 10 cases per 1 million people. Its annual incidence may be higher in elderly and hospitalized patients.^[4]^ If the platelet count drops sharply during drug use, DITP should be considered. More than 300 drugs have been associated with DITP, among which the most common psychotropic drugs known to cause thrombocytopenia are escitalopram, fluoxetine, mirtazapine, amitriptyline, sertraline, paroxetine, mianserin, and imipramine.^[3,5–7]^ In this report, we present a rare case of DITP secondary to venlafaxine treatment.

Venlafaxine is a SSRI commonly used to treat depression, anxiety, and other psychiatric disorders.^[3]^ Venlafaxine rarely causes DITP, and possible mechanisms include^[2,8–11]^: One mechanism includes directly antagonizing the serotonin transporter and inhibiting platelet aggregation leading to the reduction of serotonin levels in platelets. Serotonin is an important endogenous substance in the body, and more than 90% is stored in platelets in the peripheral circulation, which play an important role as carriers for the storage and transportation of serotonin.^[8–10]^ After vascular injury, serotonin is released from platelets to promote vascular contraction and change platelet morphology to promote their aggregation.^[11]^ Serotonin transporters are responsible for taking serotonin into platelets. Once blood vessels are damaged, serotonin is released from platelets again, promoting vasoconstriction and platelet aggregation. SSRIs inhibit this process, thereby increasing the risk of bleeding. The other is that drug-dependent antibodies mediated platelet count reduction.^[12–14]^ Drugs act as haptens and bind to plasma or platelet proteins to complete antigens to produce corresponding antibodies, which can activate the complement system, damage platelets, caused premature platelet clearance by macrophages in the reticuloendothelial system, resulting in thrombocytopenia.

Patients with DITP typically develop severe thrombocytopenia (platelets < 20 × 10^9^/L) within 5 to 10 days of exposure.^[15]^ The main clinical manifestations include petechiae, bruising, epistaxis, gastrointestinal hemorrhage, and intracranial hemorrhage. The risk of bleeding is often associated with platelet count, and up to 9% of patients with DITP die of major bleeding and rare disseminated intravascular coagulation. The diagnosis of DITP mainly depends on the patient’s history and clinical manifestations and is an exclusive diagnosis. Shah et al^[16]^ suggested that the clinical criteria for the diagnosis of DITP include the occurrence of thrombocytopenia while taking the causative drug, exclusion of other causes of thrombocytopenia after discontinuation of the drug, reuse of the causative drug causes a recurrence of thrombocytopenia, or identification of drug-dependent platelet antibodies in vitro. Drug-dependent platelet-reactive antibody detection uses flow cytometry or enzyme immunoassay to detect drug-dependent platelet-reactive antibodies. These tests can reduce diagnostic uncertainty; however, they are not commonly used in clinical practice. Our patient developed subcutaneous bleeding 8 days after taking Efflafaxine, and hematograms indicated severe thrombocytopenia. Unfortunately, the patient did not cooperate in testing for drug-dependent platelet autoantibodies. Therefore, we mainly rely on clinical presentation and laboratory data and make diagnoses according to the aforementioned criteria. In this case, evidence supporting DITP or venlafaxine-induced immune thrombocytopenia included severe thrombocytopenia 8 days after exposure to venlafaxine. Other causes of thrombocytopenia such as heparin-induced thrombocytopenia purpura, hemolytic uremic syndrome, nutritional deficiency, liver dysfunction, infection, and even hematologic neoplasms were excluded. Although the patient was exposed to venlafaxine while taking other antianxiety medications, the patient continued to take other antianxiety medications orally after his platelets returned to normal and did not develop thrombocytopenia again during a 3-month follow-up. Additionally, no studies have reported that tandospirone citrate capsules and Zolpidem tartrate tablets can cause thrombocytopenia.

In clinical practice, DITP and ITP are sometimes indistinguishable. Once DITP is considered, the suspected drug is discontinued immediately and thrombocytopenia begins to recover after 4 to 5 half-lives of discontinuation.^[15]^ Patients with severe thrombocytopenia and bleeding who are at a high risk of bleeding may be administered glucocorticoids and high-dose intravenous immunoglobulin (IVIG). Glucocorticoids cause the apoptotic death of autoantibody-producing lymphocytes, and intravenous immunoglobulin interferes with macrophage consumption of autoantibody-coated platelets.^[16]^ In addition, thrombopoietic drugs, monoclonal immunosuppressants, and other immune agents are available.^[17]^ It is important to note that as long as the drug or its metabolites are present in the plasma, platelet transfusion is usually ineffective, but has some hemostatic effect. After the exclusion of secondary factors, the patient was administered glucocorticoids combined with thrombopoietic drugs and platelet infusion treatment, and the platelet count of the patient gradually returned to normal. Thus, although we did not detect drug-dependent autoantibodies in patients, the combination of the severity of thrombocytopenia and the duration of platelet decline after the patient’s use of the suspected drug led to clinical suspicion of DITP and ultimately clinical confirmation that it was also responsive to treatment.

In summary, venlafaxine may rarely cause thrombocytopenia, and clinicians must pay attention to it in the process of using venlafaxine. Once suspected, the drug should be stopped on time and intervention measures should be taken to ensure drug safety.

## Author contributions

**Conceptualization:** Shi Lijun, Ma Zhongrui, Wei Li.

**Data curation:** Shi Lijun.

**Formal analysis:** Shi Lijun.

**Funding acquisition:** Shi Lijun, Feng Lei.

**Investigation:** Shi Lijun.

**Methodology:** Shi Lijun.

**Project administration:** Shi Lijun, Wei Li, Feng Lei.

**Resources:** Shi Lijun, Wei Li, Jiang Wei, Yu Xia, Pan Yaning.

**Software:** Shi Lijun, Pan Yaning.

**Supervision:** Shi Lijun.

**Validation:** Shi Lijun.

**Visualization:** Shi Lijun.

**Writing – original draft:** Shi Lijun.

**Writing – review & editing:** Shi Lijun.
